# Comparison of Infectious Agents Susceptibility to Photocatalytic Effects of Nanosized Titanium and Zinc Oxides: A Practical Approach

**DOI:** 10.1186/s11671-015-1023-z

**Published:** 2015-08-04

**Authors:** Janusz Bogdan, Joanna Zarzyńska, Joanna Pławińska-Czarnak

**Affiliations:** Department of Food Hygiene and Public Health Protection Faculty of Veterinary Medicine, Warsaw University of Life Sciences—SGGW, Nowoursynowska 159, 02-776 Warsaw, Poland

**Keywords:** Nanotechnology, Nanoparticles, Photocatalysis, Titanium dioxide, Zinc oxide, Reactive oxygen species, Infectious agents

## Abstract

Nanotechnology contributes towards a more effective eradication of pathogens that have emerged in hospitals, veterinary clinics, and food processing plants and that are resistant to traditional drugs or disinfectants. Since new methods of pathogens eradication must be invented and implemented, nanotechnology seems to have become the response to that acute need. A remarkable achievement in this field of science was the creation of self-disinfecting surfaces that base on advanced oxidation processes (AOPs). Thus, the phenomenon of photocatalysis was practically applied. Among the AOPs that have been most studied in respect of their ability to eradicate viruses, prions, bacteria, yeasts, and molds, there are the processes of TiO_2_/UV and ZnO/UV. Titanium dioxide (TiO_2_) and zinc oxide (ZnO) act as photocatalysts, after they have been powdered to nanoparticles. Ultraviolet (UV) radiation is an agent that determines their excitation. Methods using photocatalytic properties of nanosized TiO_2_ and ZnO prove to be highly efficient in inactivation of infectious agents. Therefore, they are being applied on a growing scale. AOP-based disinfection is regarded as a very promising tool that might help overcome problems in food hygiene and public health protection. The susceptibility of infectious agents to photocatalylic processes can be generally arranged in the following order: viruses > prions > Gram-negative bacteria > Gram-positive bacteria > yeasts > molds.

## Review

### Introduction

Some metal oxides powdered to nanoparticles (NPs) (1 < *φ* ≤ 100 nm) have been attracting much interest among scientists representing various fields of science. The main reason for this ever growing interest presents photocatalytic properties that those compounds exhibit. Photocatalytic properties of titanium dioxide (TiO_2_) were first reported in the 1970s [1] and later confirmed in a number of experiments [2–5]. At the end of the twentieth century, the studies that were going on in various research centers discovered that also other metal oxides, e.g., zinc oxide (ZnO) [6–10], after they have been powdered to the NP form, exhibit formerly undisplayed photocatalytic properties. Due to those newly discovered characteristics, TiO_2_ and ZnO in the NP form have found many new applications, e.g., as ingredients of photocatalytic layers covering various work surfaces [11]. The so-coated surfaces gain self-disinfecting and self-cleaning abilities [12, 13]. It is a result of advanced oxidation processes (AOPs) initiated by the ultraviolet (UV) radiation [14]. On surfaces coated by a thin film of photocatalyst, the inactivation of infectious agents [15, 16] and the mineralization of organic matter [17, 18] take place. Due to their virucidal [19, 20], bactericidal [21, 22] and fungicidal [23, 24] properties, photocatalytic layers are increasingly applied to coat surfaces in miscellaneous premises, such as farms, abattoirs, production halls, hospitals, and laboratories. The usefulness of photocatalysis has also been proven in water treatment [25], purification of drinking water [26], and air disinfection [27]. The researchers presume that the introduction of photocatalytic surfaces in the food industry, animal production, and health-care facilities will also be beneficial, as it will help prevent food poisonings and food contaminations, contribute towards the animal welfare, and improve the efficiency of pathogen eradication [28–30].

### Photocatalytic Properties of Titanium and Zinc Oxides

The discovery in the second half of the twentieth century that nanosized titanium dioxide (nano-TiO_2_) exhibits catalytic properties if exposed to UV radiation evoked a great interest in this substance [1] that has been growing ever since [14, 31]. The photocatalytic properties of semiconductors, to which nanosized metal oxides, such as TiO_2_ and ZnO, belong, result from their specific energetic structure. Their low-energy valence band (VB) is filled with electrons, and their high-energy conduction band (CB) is electron free. The energy difference (*Δ*E) between those bands, defined as band gap, equals the amount of energy necessary to excite an electron from VB to CB. In case of three polymorphic TiO_2_ forms, i.e., brookite, rutile, and anatase, as well as for ZnO, the width of the energy gap amounts to 2.96, 3.02, 3.20, and 3.37 eV, respectively. It is the equivalent of the electromagnetic radiation photon energy with a wavelength of *λ* < 400 nm. The photocatalytic properties of TiO_2_ and ZnO in their NP form are applied in a number of biological experiments, in which the UV radiation is used with the purpose to excite the photocatalysts. The UV radiation is commonly applied in its near-ultraviolet range (UV-A, *λ* = 315–400 nm) [32–37].

**Fig. 1:**
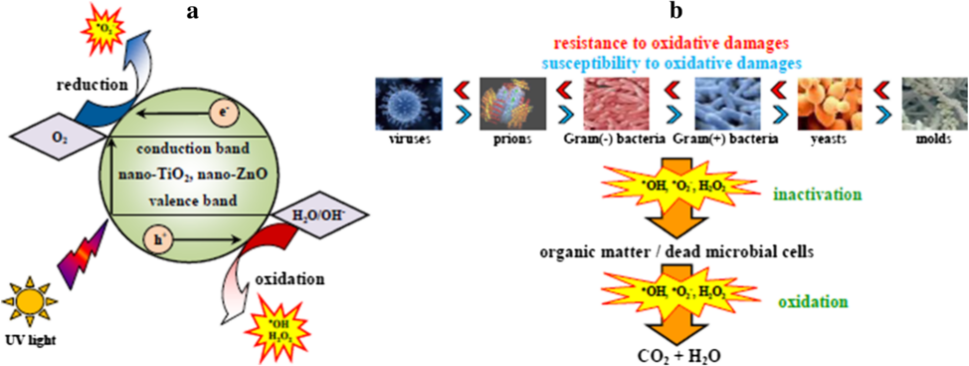
Mechanism of reactive oxygen species (ROS) generation on the surface of nanoparticles of titanium or zinc oxides (**a**) and the effects of ROS on infectious agents (**b**). On the surface of TiO_2_ and ZnO nanoparticles, exposed to UV radiation, ROS (^•^O_2_^−^, ^•^OH, and H_2_O_2_) are formed (**a**). ROS have the ability to inactivate infectious agents susceptibility of which to oxidative damages can be arranged in the following order: viruses > prions > Gram(−) bacteria > Gram(+) bacteria > yeasts > molds (**b**). Subsequently, organic matter and dead microbial cells are oxidized by ROS to CO_2_ and H_2_O (**b**)

The result of the semiconducting metal oxides irradiation is the excitation of an electron (e^−^) from VB to CB, whereby a positively charged electron hole (h^+^) emerges. Therefore, a specific “hole-electron” pair (h^+^ + e^−^), called exciton, is generated [38, 39] (Fig. 1).

The electron holes (h^+^) induce the oxidation processes while electrons (e^−^) condition the reduction processes. The electron holes (h^+^) react with water molecules (H_2_O) or hydroxide ions (OH^−^), forming hydrogen peroxide molecules (H_2_O_2_) or hydroxyl radicals (^•^OH). Electrons (e^−^) react with molecular oxygen (O_2_), forming superoxide anion radicals (^•^O_2_^−^). Therefore, various forms of reactive oxygen species (ROS) are created: H_2_O_2_, ^•^OH, and ^•^O_2_^−^ [38–40] (Fig. 1).

ROS emerging on the photocatalytic surfaces first inactivate infectious agents such as viruses, prions, bacteria, yeasts, and molds. Then, ROS oxidize the dead microbial cells [23, 24, 41], along with organic matter [18, 42], to CO_2_ and H_2_O. Due to photocatalytic properties, TiO_2_ and ZnO are applied, after powdering to NPs, in AOPs- and UV-radiation-based methods of pathogens inactivation and organic pollutants decomposition [43]. The susceptibility of infectious agents to photocatalylic processes may by miscellaneously arranged, depending upon particular studies presented by various authors. The most representative seems to be in the following order: viruses > prions > Gram-negative bacteria > Gram-positive bacteria > yeasts > molds (Fig. 1).

### Antiviral and Antiprion Activity of Nanosized Titanium Dioxide

#### Viruses Inactivation

There are studies proving that the TiO_2_/UV process where titanium dioxide (TiO_2_), after it has been powdered to nanoparticles, performs as photocatalyst, and ultraviolet (UV) radiation is an agent generating reactive oxygen species, is an effective tool in the eradication of many viruses from a number of taxonomy groups (according to the Baltimore system of virus classification) [44, 45]. Among viruses inactivated by the TiO_2_/UV process, there are: icosahedral enveloped viruses from the dsDNA group, e.g., herpes simplex virus type 1 (HSV-1) [42, 46], and from the dsDNA-RT group, e.g., hepatitis B virus (HBV) [47]; icosahedral non-enveloped viruses from the dsRNA group, e.g., rotavirus A (RV-A) [48], and from the (+)ssRNA group, e.g., poliovirus (PV) [49]; helical enveloped viruses from the (−)ssRNA group, e.g., avian influenza H5N2 virus (A/H5N2) [43]; as well as icosahedral phages of *Escherichia coli*, such as MS2 from the (+)ssRNA group [35, 50, 51] and T4 from the dsDNA group [52, 53]. In the past, air-borne virus diseases appeared to pose a serious health risk for large human populations. As an example, severe acute respiratory syndrome (SARS) can be quoted. It was detected in China in 2003, for the first time. Another example is the influenza epidemic that spread over the whole world in 2009, growing to nearly pandemic dimensions. Nonspecific character of AOPs gives rise to assume that the TiO_2_/UV process could efficiently reduce the dissemination of many viruses, e.g., measles, mumps, rubella, or smallpox. Some authors, like Han et al. [54], believe the TiO_2_/UV process can be an efficient tool to reduce the dissemination of the SARS virus, a helical enveloped virus from the (+)ssRNA group. Nakano et al. [55] showed that on the surface covered by a thin layer of nano-TiO_2_, a complete inactivation of influenza virus took place within a short period (approximately 30 min) of the TiO_2_/UV process. Those results have also been confirmed in the studies of other authors [56]. Despite the common opinion of the relatively high virus susceptibility to AOPs (Fig. 1), Josset et al. [57] and Zhao et al. [58] suggest a higher (compared to bacteria) resistance of viruses to photocatalytic processes.

**Fig. 2:**
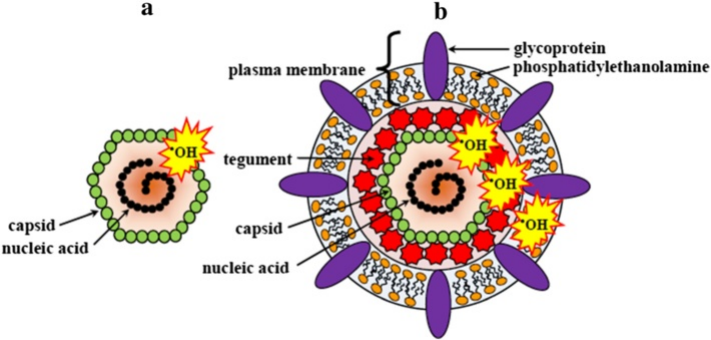
Susceptibility () of layers encircling the nucleic acid in non-enveloped (**a**) and enveloped (**b**) viruses to damages induced by hydroxyl radicals (^•^OH). Susceptibility of the various layers encircling the viral nucleic acid to oxidative damages is almost identical (plasma membrane ≈ tegument ≈ capsid)

The virus inactivation mechanism by AOPs is still insufficiently examined [59]. Kashige et al. [60] suggest the TiO_2_/UV-based inactivation of the phage PL-1 that infects *Lactobacillus casei* should be attributed mainly to the damages of capsid proteins inflicted by hydroxyl radicals and superoxide anion radicals. As the authors report, the fragmentation of the viral nucleic acid follows. The same opinion with respect to the MS2 phage infecting *E. coli* represents Kim et al. [61] as well as Sjogren and Sierka [62]. According to Liga et al. [63, 64], non-enveloped viruses are more prone to the oxidizing activity of hydroxyl radicals than enveloped viruses. Their nucleic material is separated from the external environment only by a thin layer of capsid. In enveloped viruses, the nucleocapsid, also called virion, is surrounded by a plasma membrane that protects the viruses from external factors. This envelope is formed of virus-produced glycoprotein spikes and of a phospholipid bilayer derived from the host cell and composed by phosphatidylethanolamine (PE). In all herpesviruses, there is a tegument, a protein cluster, which is located between the envelope and nucleocapsid [46]. Xu et al. [59] report that the differences between the enveloped and non-enveloped viruses in their susceptibility to photocatalytic processes result from the number of layers, hence the difference in the overall thickness, separating the virus nucleic material from the external environment, rather than from the various susceptibilities of the particular layers that surround the nucleic material (Fig. 2).

Not all authors share, however, the above outlined conclusions. Nakano et al. [65] report that the non-enveloped viruses exhibit greater resistance to the destructive activity of the TiO_2_/UV process. In their experiments, they examined the susceptibility of two viruses: an influenza virus (IFV), a representative of the enveloped viruses group, and a feline calicivirus (FCV) from the non-enveloped viruses group. Both viruses were inactivated in a time-dependent manner. It took for the inactivation of FCV to fall below the detection limit as much as twice the time as it was needed in case of IFV. The authors suggest those differences are attributed to the viral envelope. The products of peroxidation of the envelope membrane phospholipids promote the oxidative damages to capsid proteins. As a consequence, the damages to nucleic acid, hence the virus inactivation, occur faster.

#### Prions Inactivation

Hydroxyl radicals formed during the TiO_2_/UV process inactivate also protein infectious agents, so-called prions. Prions belong to the group of pathogens that exhibit a fairly high resistance to conventional disinfection methods. Paspaltsis et al. [66, 67] observed in vitro the full decomposition of the scrapie prion protein (PrP^Sc^), a protein causing one of the several transmissible spongiform encephalopathies (TSEs), after 60 min of UV-A irradiation in the presence of 0.8 % colloidal nano-TiO_2_. The researchers also reported that the decomposition of the cellular prion protein (PrP^C^), a protein not inducing any of the TSEs, took place already after 30 min of the TiO_2_/UV process. A greater resistance of PrP^Sc^ to oxidative activity of ROS is attributed to alleged differences in the protein secondary structure, a reason for the protein’s different physicochemical properties. Paspaltsis et al. [66, 67] stress that the dimensional structure of PrP^Sc^ still remains hypothetical. Due to its insolubility in water, it is impossible, though, to examine PrP^Sc^ using nuclear magnetic resonance (NMR) spectroscopy. Prion transmission, hence prion-induced diseases, constitutes a serious risk for the health of people and animals alike. The main source of infection is the contamination of surgeon tools by prions. The implementation of the TiO_2_/UV-based disinfection of medical tools and devices in the human and veterinary medicine might help reduce the number of TSEs infections on the iatrogenic pathway.

### Antibacterial Activity of Nanosized Titanium and Zinc Oxides

Contemporary human and veterinary medicine have found measures to prevent and heal many bacterial diseases, using traditional methods, such as vaccination and antibiotic therapies. Nonetheless, numerous microorganisms present in clinical and non-clinical environments have recently become much more resistant to drugs [68]. Currently, the main problem in hospitals and veterinary clinics is the presence and infestation of highly virulent and antibiotic-resistant pathogens. Among them, there are Gram(−) bacteria, e.g., carbapenem-resistant *Acinetobacter baumanii* (CRAB) [69], carbapenem-resistant *Klebsiella pneumonia* (CRKP) [70], carbapenem-resistant *Pseudomonas aeruginosa* (CRPA) [71], as well as Gram(+) bacteria, e.g., vancomycin-resistant enterococci (VRE) [72], methicillin-resistant *Staphylococcus aureus* (MRSA) [73], vancomycin-resistant *S. aureus* (VRSA) [74], methicillin-resistant *Staphylococcus epidermidis* (MRSE) [73], or penicillin-resistant *Streptococcus pneumoniae* (PRSP) [75]. Thus, the search for effective methods of pathogen eradication presents a crucial challenge for many research centers in the world [21, 22, 76]. One of the latest achievements in this field has been the application of photocatalysis [77].

#### Titanium Dioxide

Nano-TiO_2_ exhibits not only virucidal properties. The TiO_2_/UV process is a tool for an efficient bacteria eradication, too [77–83]. Its antibacterial activity depends upon such factors as light intensity [2], concentration and diameter of photocatalyst particles [10, 84], temperature of environment [3], chemical composition of the base [85, 86], and sensitivity of microorganism species [87, 88]. According to Ferin and Oberdörster [89] as well as Hwang et al. [90], the highest photocatalytic activity, hence the strongest bactericidal properties are shown by two polymorphic forms of nano-TiO_2_, anatase, and rutile, combined in the proportion 80 and 20 %, respectively. This composition is widely used in experiments with bactericidal properties of nano-TiO_2_ and is commonly known under the trade name P-25 [91]. Some literature reports that both forms (anatase and rutile) are equally harmful to bacteria [10]. However, most authors report that anatase shows stronger catalytic properties than rutile [92]. Various studies examined the optimal concentration of P-25 in aqueous solutions for the purpose of bacteria eradication. Liou and Chang [93] as well as Pigeot-Rémy et al. [94] prove that, irrespectively of the initial density of *E. coli* cells (10^2^–10^8^ CFU mL^−1^), the highest bacteria inactivation rate was observed at 0.1 % P-25. Other authors concentrated on the influence of the diameter of nano-TiO_2_ particles on its bactericidal properties. Salih and Pillay [95], as well as many other scientists [96, 97], came to the conclusion that nano-TiO_2_ with *φ* < 20 nm has stronger bactericidal properties than its less powdered form. It can be attributed to the fact that particles with *φ* < 20 nm are able to penetrate the damaged cell envelope (cell wall and plasma membrane) and infiltrate the cytosol while particles with *φ* = 20–80 nm are not able to overcome the barrier of a cell envelope. The nano-TiO_2_ particles’ antibacterial activity increases as their size decreases (surface-area-to-volume ratio). The smaller the NP size, the bigger the surface area of the nano-TiO_2_ particles, hence the number of ROS. Other studies point out that environmental factors affect the efficiency of the TiO_2_/UV process. Tong et al. [86] indicate that various factors, including organic pollutants, may cause a decrease of bacteria inactivation rate, even by 40 %. Results of other experiments suggest the underlying factor to weaken the nano-TiO_2_ photocatalytic effect might also be the high density of microorganism cells (in excess of 10^8^ CFU mL^−1^) [2, 85, 98].

**Fig. 3:**
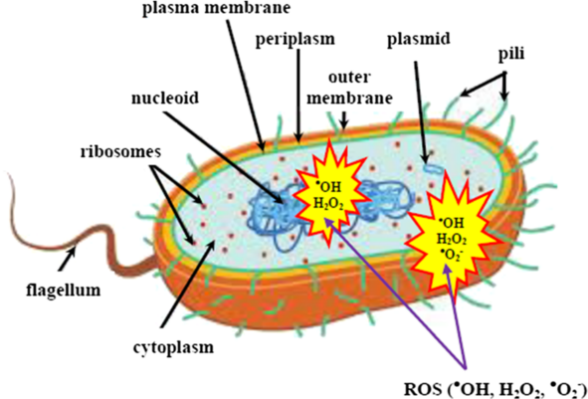
Bacteria cell structures most easily damaged by reactive oxygen species (ROS). Among the bacteria cell structures, cell envelope (cell wall and plasma membrane) as well as nucleoid are most affected by ROS-induced damages. Hydroxyl radicals (^•^OH) and hydrogen peroxide molecules (H_2_O_2_) have the ability to penetrate the bacteria cell envelope, whereas superoxide anion radicals (^•^O_2_^−^) do not exhibit such ability

The studies on the susceptibility of bacteria to the TiO_2_/UV process date back to 1985 when Matsunaga et al. [4] for the first time reported that a 120-min exposition of water polluted by *E. coli* and *Lactobacillus acidophilus* to the UV-A radiation resulted in an almost complete destruction of the bacteria. This experiment used photocatalytic properties of the nanosized titanium dioxide loaded by platinum (nano-TiO_2_/Pt). The NPs’ addition of some chemical elements, both metals (e.g., Ag, Au, and Pt) and non-metals (e.g., C, N, and P), as well as their oxides (e.g., WO_3_ and CrO_3_), results in the broadening of the electromagnetic radiation spectrum capable of exciting electrons in a semiconductor, e.g., nano-TiO_2_, and thereby enhances its effectiveness as a photocatalyst [5, 99]. In the inactivation of bacteria by the TiO_2_/UV process, ROS plays a crucial role. The strongest bactericidal activity among the ROS is attributed to hydroxyl radicals [14, 18, 36, 38, 100]. Cho et al. [101] claim there is a linear correlation between the *E. coli* inactivation rate and the hydroxyl radical concentration in bacteria cells. Bekbölet and Araz [102] as well as Salih [103] maintain that high oxidation potential and nonspecific reactivity of hydroxyl radicals are the main factors of their bactericidal properties. Hydroxyl radicals (^•^OH) are short living, particularly unstable and react rapidly with most biological molecules [40]. They can penetrate the cell wall, oxidize membrane fatty acids, induce lipid peroxidation, oxidize proteins, and damage DNA. Proteins are affected by tyrosine hydroxylation, oxidation of methionine or cysteine, as well as by the carbonyl group formation on side-chain amino acids [104, 105]. Similar effects are induced by hydrogen peroxide molecules (H_2_O_2_) [36, 106, 107] (Fig. 3).

During the oxidation of the bacteria cell envelope phospholipids, carbonyl compounds such as aldehydes, ketones, and carboxylic acids are formed. Some of them exhibit properties that are potentially toxic to bacteria [16, 108]. Liu et al. [105], as well as other authors [109, 110], observed at the model of *E. coli* that various layers of the bacteria cell envelope are characterized by a different susceptibilities to hydroxyl radicals generated as a result of photocatalytic processes, e.g., the TiO_2_/UV process. According to those authors, the highest resistance was shown by peptidoglycan (PG), also called murein. In Gram-positive bacteria, the PG provides for an efficient physical barrier that prevents from the entry of molecules into the cell. Much more susceptible to oxidative damages was PE that builds a phospholipid bilayer of plasma membrane, while lipopolysaccharides (LPS), present exclusively in outer membrane of Gram-negative bacteria cell wall, submitted particularly easy to the oxidative damages. Porins going across the outer membrane facilitate small molecules to enter the cells. In bacteria exposed to the TiO_2_/UV process, significant morphological changes in the cell structure were observed, using the scanning electron microscopy (SEM), transmission electron microscopy (TEM), and atomic force microscopy (AFM) [111, 112]. Various cell damages were noticeable, e.g., plasmolysis, intracellular vacuoles ghost, cell debris, and nucleoid condensation [113, 114]. In some cases, a separation of the plasma membrane from the PG layer occurred. As a general rule, the bacteria inactivation progressed with no visible PG degradation, neither with Gram-negative nor with Gram-positive bacteria [115]. Hydroxyl radicals (^•^OH), generated during the TiO_2_/UV process, infiltrate the cell envelope, subsequently disrupting nucleic acids. They destroy phosphodiester linkages, induce the formation of pyrimidine dimers [32, 100, 116, 117], or lead to a complete oxidation of purine and pyrimidine bases to CO_2_, H_2_O, and NH_3_ [17, 18]. Hydroxyl radicals can also damage the DNA of bacterial plasmid [118] and induce the dimerization of coenzyme A (CoA) particles [119]. The CoA is the intracellular carrier of acyl groups and electrons. Its depletion disrupts the process of oxidative pyruvate decarboxylation and the Krebs cycle, both important stages of glucose catabolism. The increase in the concentration of the dimeric CoA inhibits the cell respiration. Imlay et al. [120], similarly Ndounla et al. [121], emphasize that the formation of bactericidal hydroxyl radicals does not occur exclusively as a consequence of nano-TiO_2_ excitation by UV radiation. As they report, a simultaneous process is the Fenton reaction. There, the formation of hydroxyl radicals (^•^OH) is actively enhanced by Fe^2+^ ions and hydrogen peroxide molecules (H_2_O_2_) (see Eq. 1).

1 $$ {\mathrm{Fe}}^{2+} + {\mathrm{H}}_2{\mathrm{O}}_2\to\ {\mathrm{Fe}}^{3+}{+}^{\bullet}\mathrm{O}\mathrm{H} + {\mathrm{O}\mathrm{H}}^{-} $$

Superoxide anion radicals (^•^O_2_^−^) cannot penetrate the cell envelope since they are negatively charged. However, contrary to hydroxyl radicals, they show a relatively long living period [36] (Fig. 3).

Bacteria, similar to cells of eukaryotic organisms, have also developed their own antioxidant enzyme systems, i.e., mechanisms protecting them from ROS. The principal defense mode is a system of three enzymes that are responsible for detoxification of ROS. These are: superoxide dismutase (SOD; EC 1.15.1.1), a protein catalyzing the disproportionation reaction of two superoxide anion radical particles to hydrogen peroxide and molecular oxygen and catalase (CAT; EC 1.11.1.6) and glutathione peroxidase (GPX; EC 1.11.1.9), enzymes decomposing hydrogen peroxide into water and molecular oxygen [122, 123]. The crucial role in limiting the negative effects of oxidative stress plays also as antioxidants such as lipoic acid (LA) and ubiquinone (CoQ_10_). LA is considered to be an important endogenous free radical scavenger as it neutralizes free radicals in lipid and aqueous domains alike. CoQ_10_ performs also as an energy carrier. Kim et al. [124] and Ojima et al. [125] confirmed in their experiments with *E. coli* that in case the ROS concentration is too high for the defense systems to repair, numerous oxidative damages of the nucleoid and cell envelope phospholipids domain occur. The products of polyunsaturated fatty acids (PUFAs) peroxidation, such as malondialdehyde (MDA), can form adducts with nucleic acids and proteins thus, leading to alterations in their functioning. The DNA damages result in various mutations, while the protein injuries cause enzymes inhibition, denaturation and protein degradation. As those alterations become excessive, they may eventually lead to the cell death. Simon-Deckers et al. [10] studied other Gram-negative bacteria, *Cupriavidus metallidurans*, and stated that the cell death is, on the one part, a result of the amount of ROS and the extent of the damages caused by them; and, on the other part, a result of the ability to keep the cell envelope integrity by the intracellular repair systems.

Sunada et al. [126] represent the opinion that the bacteria inactivation during the TiO_2_/UV process takes place in three steps. At first, oxidative damage of the outer membrane occurs, causing merely insignificant impairment to bacteria viability. Thereafter, a DNA injury, the lowering of CoA level, increased permeability of the plasma membrane and the leakage of the intracellular components follow. Finally, the microorganisms die.

**Fig. 4:**
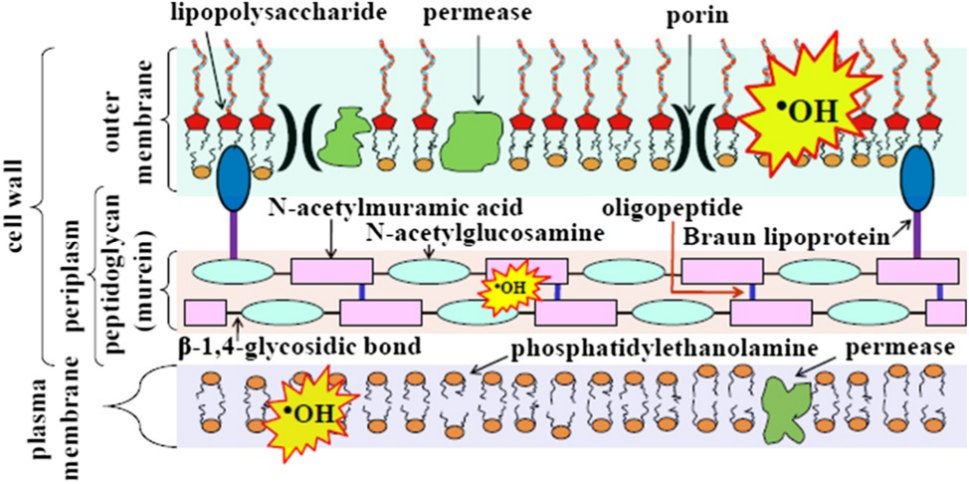
Susceptibility () of Gram-negative bacteria cell envelope compounds to damages induced by hydroxyl radicals (^•^OH). Susceptibility of the various compounds of the Gram(−) bacteria cell envelope (cell wall and plasma membrane) to oxidative damages presents the following order: lipopolysaccharides > phospholipids > peptidoglycan

The differentiation in the bacteria susceptibility to the TiO_2_/UV process results from two factors: various structure of the cell wall (its complexity and thickness) and different susceptibilities of the cell wall compounds to oxidative damages. Bactericidal effects that the TiO_2_/UV process has on many microorganisms have been confirmed. Moderate resistance against oxidative damages was exhibited by the Gram-negative bacteria, e.g., *E. coli* [2, 5, 33, 34, 79, 86, 98, 99, 105, 119, 124, 127–133], *K. pneumoniae* [134], *Salmonella* Enteritidis [37, 135], *Salmonella* Typhimurium [127], *Serratia marcescens* [136], *Shigella flexnerii* [15, 127], *Legionella pneumophila* [137], *A. baumannii* [15, 128, 136], *P. aeruginosa* [34, 37, 77, 78, 129, 138, 139], and *Vibrio cholerae* [127]. Gram-negative bacteria have a fairly thin cell wall (2–10 nm) which is formed of two phospholipid bilayers (outer membrane and plasma membrane), separated by periplasmic space in which two to three PG layers are immersed. Murein is a net of long, unbranched chains formed of particles of n-acetylglucosamine (GlcNAc) and n-acetylmuramic acid (MurNAc) that are alternately arranged and linked by β-1,4-glicosidic bond. The chains are bound by transversal (horizontal and vertical) oligopeptide bridges. Unlike the plasma membrane, which is formed almost exclusively of PE, the outer membrane consists of two asymmetrically placed lipid layers. The inner layer is formed of PE, and the outer layer of LPS that in pathogens play the role of endotoxins and are a significant pathogenic agent [105, 108, 110] (Fig. 4).

**Fig. 5:**
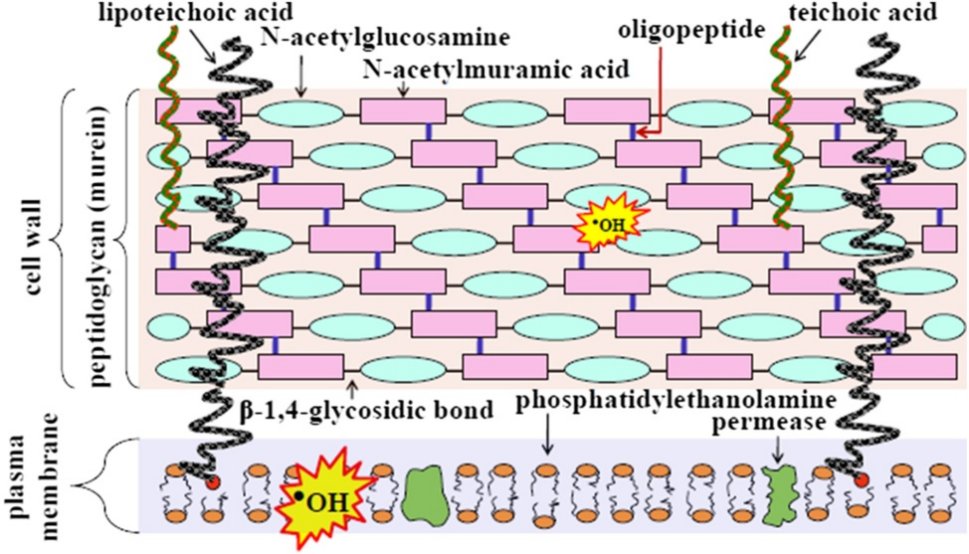
Susceptibility () of Gram-positive bacteria cell envelope compounds to damages induced by hydroxyl radicals (^•^OH). Susceptibility of the various compounds of the Gram(+) bacteria cell envelope (cell wall and plasma membrane) to oxidative damages presents the following order: phospholipids > peptidoglycan

A greater resistance to the destructive properties of ROS show Gram-positive bacteria species, e.g., *S. aureus* [15, 34, 76, 129, 140], *Bacillus anthracis* [141–144], *Bacillus cereus* [145], *Bacillus pumilis* [146], *Bacillus subtilis* [33, 141], *L. acidophilus* [4, 147], *Lactobacillus helveticus* [130], *Clostridium perfringens* [148], *Enterococcus faecalis* [149], *Enterococcus faecium* [34], and *Listeria monocytogenes* [78, 136, 150, 151]. Gram-positive bacteria have a relatively thick cell wall (20–80 nm). Their plasma membrane is formed similarly as in Gram-negative bacteria and is surrounded by around 40 layers of PG. Long, polymeric, and sticking over the surface of cell wall chains of teichoic acids pass across murein. Lipoteichoic acid (LTA) is rooted in plasma membrane whereas teichoic acid (TA) is bound to polysaccharide murein chains (more precisely, to the groups of MurNAc) [108, 110] (Fig. 5).

As Markowska-Szczupak et al. [152] report, the bacterial susceptibility to the TiO_2_/UV process can be ordered as follows: *E. coli* > Gram-negative bacteria (other than *E. coli*) > Gram-positive bacteria (other than *Enterococcus* sp.) > *Enterococcus* sp. (Fig. 1). This sequence is confirmed also by Kühn et al. [34] who arranged the inactivation effectiveness of four bacteria species on a surface covered by a thin layer of nano-TiO_2_ and exposed to UV-A radiation during 60 min as follows: *E. coli* > *P. aeruginosa* > *S. aureus* > *E. faecium*. The experiments conducted by Hitkova et al. [153] confirm that Gram-negative bacteria are more susceptible to the photocatalytic inactivation than Gram-positive bacteria. The entire reduction of *E. coli* and *P. aeruginosa*, two Gram-negative bacteria representatives, was achieved within 15 and 25 min, respectively, while it took 35 min to completely inactivate *S. aureus*, a Gram-positive bacteria species. A great number of studies confirm the above outlined susceptibility sequence [62, 78, 81, 101, 154–160]. There are, however, authors who report the opposite order [4, 161–163]. According to them, the Gram-negative bacteria are more resistant to oxidative damages. Nakano et al. [65] examined the period needed to entirely inactivate *S. aureus*, a Gram-positive bacteria. It appeared to be four times shorter than that needed to destroy *E. coli* and *S. marcescens*, two Gram-negative bacteria species. Kubacka et al. [164] and Wolfrum et al. [24] reported there was no difference in sensitivity between the Gram-negative and Gram-positive bacteria.

Numerous studies confirm the high effectiveness of TiO_2_/UV process in eradication of methicillin-resistant *S. aureus* (MRSA) [73, 165, 166] and of UV-resistant *Enterobacter cloacae* [167]. Hydroxyl radicals that are formed during the TiO_2_/UV process have high oxidation potential, and they can also eradicate spores of *B. anthracis* and *B. cereus* [141–143].

#### Zinc Oxide

The nanosized zinc oxide (nano-ZnO) exhibits strong bactericidal properties when exposed to the UV radiation. Therefore, the ZnO/UV process presents another example of a tool that provides for an efficient bacteria eradication. The inactivation is effective for many species of Gram-negative (e.g., *E. coli*, *L. monocytogenes*, *S.* Enteritidis) and Gram-positive bacteria (e.g., *B. subtilis*, *S. aureus*, *Streptococcus pyogenes*, *E. fecalis*) [6–9, 78, 130, 168–171]. According to some authors [7, 8, 172, 173], bactericidal effects of nano-ZnO are directly and inversely proportional to the concentration and diameter of its particles, respectively. Having used the confocal laser scanning microscopy (CLSM), Raghupathi et al. [172] concluded that the accumulation of nano-ZnO in the cytoplasm or on the outer membrane might be involved in the antibacterial activity of nano-ZnO. Yamamoto [8] and Zhang et al. [9] emphasized that in the inactivation of bacteria by the ZnO/UV process, the crucial role played hydroxyl radicals and hydrogen peroxide molecules with their ability to infiltrate through the cell wall. The studies of Azam et al. [78] as well as Liu and Yang [130] showed that PE, the main component of the cell wall of Gram-negative bacteria, was much easier to penetrate by hydroxyl radicals and much more susceptible to oxidative damages than murein, the main component of the cell wall of Gram-positive bacteria. Thus, the cell wall of *E. coli* (Gram-negative bacteria) was easier to penetrate and damage by hydroxyl radicals and hydrogen peroxide molecules than the cell wall of *S. aureus* (Gram-positive bacteria) [6, 9, 78, 130]. In their experiments with *Campylobacter jejuni*, a Gram-negative bacteria, Xie et al. [174] observed that this very common food-borne pathogen was very sensitive to the ZnO/UV process. The overall growth impairment rate appears, therefore, clearly higher in case of Gram-negative bacteria. This conclusion was, however, not confirmed by Jain et al. [175] and Siddique at al. [173] who reported that nano-ZnO stronger restricts the growth of Gram(+) bacteria than Gram(−) bacteria.

Gordon et al. [168] examined the bacteria inactivation rate by superoxide anion radicals. According to them, negatively charged superoxide anion radicals did not infiltrate through the strongly negatively charged cell wall of *E. coli* as easily as through *S. aureus* cell wall which is formed mainly of PG, hence exhibits a lower density of the negative charge. This characteristic explains, as the authors say, the higher susceptibility of Gram(+) bacteria to superoxide anion radicals than that of Gram(−) bacteria.

As a matter of fact, the results of a great majority of experiments prove a higher susceptibility of Gram-negative bacteria (e.g. *E. coli*) to the ZnO/UV process than that of Gram-positive bacteria (e.g. *S. aureus*) [6, 9, 78, 130]. Such a difference in susceptibility is attributed to the different chemical composition and structure of the bacteria cell wall. Gram(−) bacteria have a much thinner cell wall compared to Gram(+) bacteria, and the content of PG, a compound relatively resistant to oxidative damages, in their cell wall is quite low.

### Antifungal Activity of Nanosized Titanium and Zinc Oxides

Fungi play an important role in our everyday life. For instance, they influence the quality of the air in rooms. Allergies, asthma, or respiratory tract infections are just some of a great number of ailments conditioned by the presence of fungi in closed areas. Pollutions in buildings are often a factor inducing the so-called sick building syndrome [176]. Many researchers believe that AOPs taking place on surfaces covered by a thin layer of photocatalyst, such as nano-TiO_2_ or nano-ZnO, might be a tool to effectively restrict the dissemination of yeasts and molds, just as it is the case with viruses and bacteria harmful to humans and animals [4, 24, 80–83, 142].

### Yeasts Inactivation

**Fig. 6:**
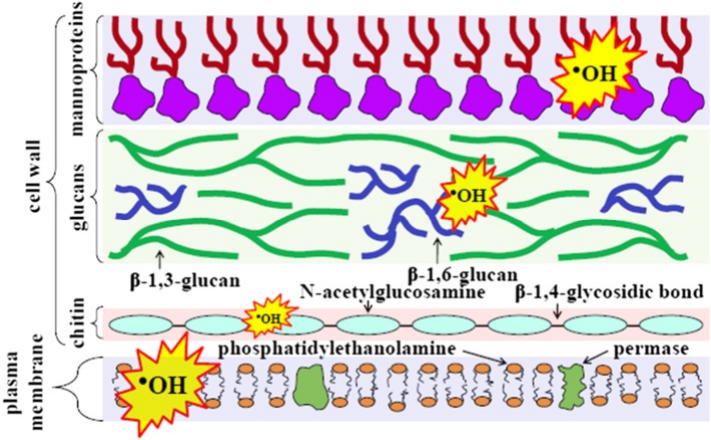
Susceptibility () of yeasts cell envelope compounds to damages induced by hydroxyl radicals (^•^OH). Susceptibility of the various compounds of the yeasts cell envelope (cell wall and plasma membrane) to oxidative damages presents the following order: phospholipids > glycoproteins > glucans > chitin

Pioneering studies evaluating the effectiveness of the TiO_2_/UV process in eradication of yeasts were conducted by Matsunga et al. [4] in 1985. They proved that the number of inactivated in vitro cells of *Saccharomyces cerevisiae* after 240 min of UV-A irradiation increased from 72 % to nearly 98 % as a result of application of 0.5 % colloidal nano-TiO_2_. Thabet et al. [177] reported there was no presence of nano-TiO_2_ particles in *S. cerevisiae* cells despite a long exposition of the pathogen to the TiO_2_/UV process, even though of the ROS concentration in cytosol increased. Those results suggest the damages in the cell wall and in the plasma membrane were merely insignificant. Fungicidal properties of nano-TiO_2_ have been also confirmed at the example of another representative of yeasts, *Candida albicans* [23, 80–82, 178, 179]. The growth of this pathogen was strongly impaired also in the presence of nano-ZnO [180–183]. According to Gondal et al. [181], the minimal inhibitory concentration (MIC) of nano-ZnO against *C. albicans* is 2.5 mg mL^−1^, a value nearly ten times higher than in case of bacteria. In both yeasts species, *S. cerevisiae* and *C. albicans*, vegetative forms exhibited a significantly lower resistance to AOPs compared to spores [80]. Lipovsky et al. [184] attribute the key role in the pathogen cells inactivation to ROS, particularly to hydroxyl radicals as agents that induce the cell envelope damages. Results of numerous studies have shown a lower susceptibility of yeasts to photocatalytic processes in comparison to bacteria [80–83, 87] (Fig. 1). Those findings have also been confirmed by the examination of their cell wall morphology, using the TEM and the X-ray diffraction (XRD) [183]. This reaction is attributed to the differences in the cell wall composition between those microorganisms [185]. Insofar the bacteria cell wall contains PG, a compound that is highly resistant to oxidative damages [105, 108, 110], the fungi cell wall contains instead a compound called chitin, which is even more resistant to the oxidative ROS activity [23]. Chitin is an unbranched polysaccharide formed of 100–160 groups of GlcNAc, linked by β-1,4-glycosidic bonds. In yeasts, this polysaccharide is present in deeper layers of the cell wall and amounts to only 1–3 % of its dry matter. The main component of the yeast cell wall is polysaccharides composed of d-glucose monomers, called glucans. In respect of their resistance to hydroxyl radicals, glucans only slightly lag behind chitin. In yeasts, mainly β-glucans are represented. A moderately branched β-1,3-glucan prevails, accompanied by much a more branched, less present β-1,6-glucan. The external layer of the yeasts cell wall is formed predominantly from mannoproteins, fairly susceptible to oxidative damages protein-sugar compounds, constituting around 40 % of cell wall dry matter. Plasma membrane formed mainly from PE and contiguous with cytosol shows the greatest susceptibility to oxidative injuries [186, 187] (Fig. 6).

#### Molds Inactivation

Recent studies prove that nanosized metal oxides, such as nano-TiO_2_ or nano-ZnO, can be applied to inactivate many species of molds (filamentous fungi), e.g., *Fusarium oxysporum* [83, 188], *Aspergillus niger* [81, 82, 181, 189], and *Penicillium expansum* [190, 191].

Chen et al. [192] reported that molds exhibit a lower resistance to the TiO_2_/UV process than viruses, bacteria, or even yeasts (Fig. 1). The scientists conducted an experiment with *A. niger*, growing on wood covered by a thin layer of nano-TiO_2_. It took as much as 12 h to stop the growth of the *A. niger* hyphae, using the UV-A radiation. Even then, the fungus spores remained viable. The long UV-A irradiation period was not able to inactivate the spores. After the source of UV-A radiation had been removed, the germination of spores and the growth of hyphae were noticed. The studies of Yu et al. [189] report that the AOP-based water disinfection was not able, either, to destroy the spores of *A. niger*. Despite the antifungal capability of the TiO_2_/UV process, the subsequent hyphae re-growth indicated that the photocatalytic disinfection was not sufficient to completely inactivate *A. niger* but merely managed to suppress its growth.

**Fig. 7:**
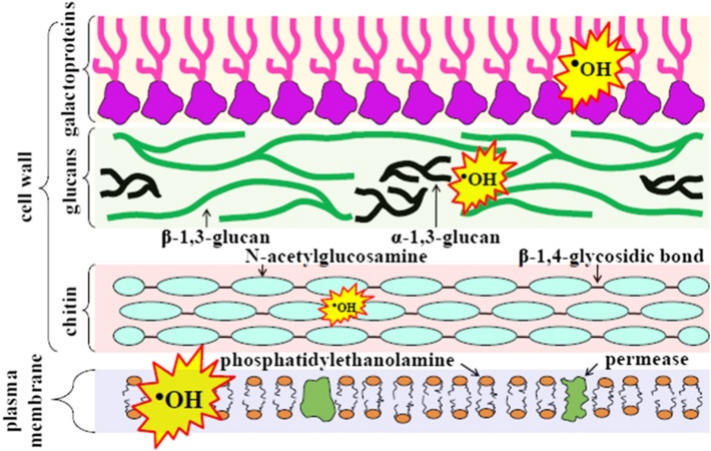
Susceptibility () of molds cell envelope compounds to damages induced by hydroxyl radicals (^•^OH). Susceptibility of the various compounds of the molds cell envelope (cell wall and plasma membrane) to oxidative damages presents the following order: phospholipids > glycoproteins > glucans > chitin

In experiments of Sichel et al. [83, 188], the spores of another representative of molds, *F. oxysporum*, were inactivated not sooner than after 10 h of the TiO_2_/UV process. Similar results in respect of this species obtained Lonnen et al. [80] as well as Mitoraj et al. [81]. The SEM, TEM, AFM, and XRD examination of *A. niger* and *F. oxysporum* cell walls showed that the spores were rugged and wrinkled. There were, however, no traces of nano-TiO_2_ presence in the spores [112]. Filamentous fungi (particularly their spores) have a fairly high resistance also towards the ZnO/UV process [181, 189, 190]. The underlying factor of the high resistance to photocatalytic processes that molds exhibit is a significant concentration of chitin in their cell wall, up to 15 % of dry matter [186, 187]. This polysaccharide protects directly the plasma membrane, which is very susceptible to the oxidative activity of ROS. A layer of glucans, thinner than in yeasts, covers a relatively thick chitin layer. The glucans are quite resistant to oxidative damages, even though they are slightly more susceptible than chitin. The main compound of the glucans in mold cell wall is β-1,3-glucan, along with a much branched α-1,3-glucan, not present in *Saccharomycetaceae*. The external layer of the molds cell wall consists of galactoproteins, protein-sugar compounds that constitute around 45 % of cell wall dry matter and exhibit less resistance to oxidative injuries than chitin and glucans [186, 187] (Fig. 7).

## Conclusions

Nanotechnology is a relatively young discipline of science. Its rapid development in recent years opens up new opportunities for many areas of human activity. Numerous fields of science, including IT, mechanical engineering, medicine, and many others, are already profiting from its achievements. Nanotechnology may largely contribute towards the fight against many pathogens, thus it may be successfully applied in such areas as food hygiene or public health protection. Infectious agents pose a threat to human health, therefore, an effective disinfection of health-care devices and surfaces appears vital for preventing the pathogens to disseminate. A reduction of infectious agent transmission in the public space can be achieved thanks to the photocatalytic properties of self-disinfecting and self-cleaning surfaces. Their crucial compounds are oxides of some metals, such as TiO_2_ and ZnO, which, after they have been powdered to NPs, exhibit strong virucidal, bactericidal, and fungicidal properties. The underlying factor hereto is photocatalytic processes induced by the UV radiation. Therefore, metal oxides coatings can become a new, powerful tool in the fight against infectious diseases. The so far commonly applied conventional chemical and pharmaceutical compounds do not provide for such a broad range of application. Nanomaterials, even though a number of them are already in use, are still not sufficiently examined in respect of their entire spectrum of properties, including possible negative side-effects on human health. Thus, the studies on innovative solutions in nanotechnology must continue. As many say, the twenty-first century fight against pathogens is going to be led primarily not by microbiologists but by nanotechnology engineers.
